# Gender Differences in Physical Fitness Characteristics in Professional Padel Players

**DOI:** 10.3390/ijerph18115967

**Published:** 2021-06-02

**Authors:** Francisco Pradas, Alejandro Sánchez-Pay, Diego Muñoz, Bernardino J. Sánchez-Alcaraz

**Affiliations:** 1 ENFYRED Research Group, Faculty of Health Sciences and Sport, University of Zaragoza, 50009 Huesca, Spain; franprad@unizar.es; 2 Human Performance and Sports Science Laboratory, Faculty of Sport Sciences, University of Murcia, 30720 San Javier, Spain; 3 Department of Musical, Plastic and Corporal Expression, Faculty of Sport Sciences, University of Extremadura, 06006 Badajoz, Spain; diegomun@unex.es; 4 Department of Physical Activity and Sport, Faculty of Sport Sciences, University of Murcia, 30720 San Javier, Spain; bjavier.sanchez@um.es

**Keywords:** physical fitness, racket sport, exercise evaluation, padel

## Abstract

The aims of the present study were to examine the fitness characteristics of professional padel players and to determine differences in physical performance regarding players’ gender. Thirty professional padel players (men: *n* = 15, age = 27.4 ± 6.8 years, height = 177.9 ± 4.0 cm; women: *n* = 15, age = 30.0 ± 4.2 years, height = 166.6 ± 4.8 cm) completed a 4-day evaluation process, including: isometric handgrip strength, sit and reach, 10 × 5 shuttle test, countermovement jump (CMJ), squat jump (SJ), Abalakov test, one-repetition maximum test (bench press, leg extension, leg curl, lat pulldowns, overhead press, and shoulder press), anthropometry and VO_2_ max tests. The men players had higher values in terms of weight, height, one maximum repetition, jump tests (CMJ and ABK) and VO_2_ max test than the women (*p* < 0.005). By contrast, the women had higher values for fat mass (*p* = 0.005; ES: 2.49). The values from this multifaceted test battery can be a useful guide for coaches regarding players’ development in future evaluations and monitoring.

## 1. Introduction

Padel is a racket sport played in pairs, which uses tennis rules and the tennis scoring system, and takes place in a rectangular area (10 × 20 m) divided into two halves by a central net, and enclosed in a synthetic glass and metal court, which allows for the use of side and back walls [1]. In recent years, a great increase in the number of participants of different genders and ages has been observed [2]. In addition, this growth has also been reflected in a greater professionalisation and an increase in scientific research [3]. Research has been focused on three fundamental aspects: temporal structure [4,5,6], players’ movements and distance covered on the court [7,8] and game actions, such as technical or tactical parameters [3,9,10].

Padel can be defined as an intermittent sport, which combines short periods of high intensity and frequent actions (0.7–1.5 per second), with alternating rest periods, as established by the game rules (20 s) [4,11]. The phosphagen system (ATP-Pc) is the main path used to obtain energy during the high-intensity actions demanded [7]. On the other hand, the maximal values of lactate obtained during the game were near to 2.4 mmol/L, indicating that anaerobic glycolysis could be less determinant [12]. Finally, the aerobic system could be relevant because of the long duration and mean intensity of the matches, requiring the complex interaction of several physical components (i.e., acceleration, changes in direction, jumps, strength), and metabolic pathways (anaerobic and aerobic) [13]. The strength and conditioning profile in padel includes short-distance sprints and changes in direction, upper-body strength and power, intermittent recovery endurance and body balance evaluations [13,14]. Due to the importance of muscular power in the game of padel, strength and power testing has become an important monitoring and evaluation tool to optimise the neuromuscular performance factors related to the major strokes [15]. The padel physical demands and workload reported, using heart rate, speed of movement and distance covered, appear to be beneficial for health promoting purposes; nevertheless, intensity patterns and fitness characteristics may change according to players’ performance levels [16].

Despite its usefulness, there is an alarming lack of investigations examining physical fitness testing in padel [13]. This information allows coaches to assess the strengths and weaknesses of players and to identify the most relevant factors in game performance [17,18]. Regarding the fitness and anthropometric characteristics of non-professional padel players, Courel-Ibáñez and Herrera-Gálvez [13] showed that men padel players had good levels of cardiorespiratory fitness, upper body power, handgrip strength, speed and agility. However, players’ tests of dynamic balance showed low values in both posterior and anterior directions. In another study, Courel et al. [19] showed that adult women that practised padel had higher levels of physical fitness than sedentary controls, due to their better body balance and explosive power, abdominal endurance, and cardiovascular capacity. Furthermore, they obtained lower waist and hip circumferences and thigh skinfold thicknesses compared to sedentary subjects.

In tennis, several sports-science and coaching staffs are regularly conducting test batteries combining general and specific tests for speed, agility, strength, power, endurance, musculoskeletal fitness, coordination and skill performance in both men and women players [18]. The optimal interpretation of these data is subsequently used in short- and long-term requirements to ensure the best possible preparation, but also to track players’ progress, creating individual profiles and detecting injury risk [18,20]. Additionally, these investigations are gaining in interest given their general applicability, replicability and affordability [21]. Thus, the aims of the present study were (1) to examine the fitness characteristics of professional padel players and (2) to determine differences in physical performance regarding players’ gender.

## 2. Materials and Methods

This analysis was conducted on data collected during a national players’ meeting using a battery of standard anthropometric and physical performance tests.

### 2.1. Participants

Thirty professional padel players (men, *n* = 15, women, *n* = 15) agreed to take part in this study. Both the men and women players were classified in the top 50 of the World Padel Tour (WPT) ranking. A summary of participants’ characteristics (anthropometric, training information, etc.) is presented in Table 1. All the participants were informed about the purpose of the study and signed a consent form before enrolling. The protocol was reviewed and approved by the Clinical Research Ethics Committee of the Department of Health and Consumption of the Government of Aragon (Spain) (21/2012). The sample was recruited by convenience, with a minimum sample size of 30 padel players (confidence level = 90%; error range = 10%).

### 2.2. Procedure

Testing protocols were conducted on four non-consecutive days with a minimum rest of 72 h between days. The tests were performed as follows: day one, isometric handgrip strength, sit and reach, 10 × 5 shuttle test; day two, countermovement jump, squat jump and Abalakov test; day three, one-repetition maximum test; day four, anthropometry and VO_2_ max test. To ensure standardisation of test administration, all tests were performed in the same order, using the same testing devices, measurement protocols and operators. The test sessions were performed in a laboratory. There was previous familiarisation with accurate testing procedures. A specific dynamic warm-up routine was carried out before the tests, consisting of joint movements, dynamic stretching, hopping exercises and jumps of increasing intensity. All the subjects were required to avoid ingesting caffeine or other types of stimulating substances, as well as intensive work sessions, from 48 h prior to the measurements.

### 2.3. Measurements

Anthropometry. Anthropometric data were collected from all participants including height using a fixed stadiometer (±0.1 cm; Seca 220, Seca, Hamburg, Germany), and weight using digital scales (±0.1 kg; Seca 714, Seca, Hamburg, Germany).

Fat mass. Anthropometric measurements were collected by the same experienced evaluator using specific equipment including a skinfold calliper (Holtain Ltd., Crymych, UK) accurate to the nearest 0.2 mm. Eight skinfold thicknesses (biceps brachii, triceps, subscapular, iliac crest, supraspinal, abdominal, thigh, and medial calf), were measured. Body mass index (BMI) was calculated from the body mass (kg) and height (m^2^) relationship. Body fat percentage was calculated using the body density formula by Withers et al. (1987) [22], estimating the percentage of body fat with the Siri equation (1961) [23].

VO_2_ max. An incremental test was performed on a treadmill in the laboratory to determine VO_2_ max, (Pulsar HP, Cosmos, Nussdorf, Germany). After a warm-up of 3 min at 6 km·h^−1^, the test was carried out with a 1% incline, starting from a speed of 8 km·h^−1^ and with increments of 1 km·h^−1^ every minute until exhaustion. The collection of expired gases was carried out with an Oxycon Pro analyser (Jaegger, Germany).

One-Repetition Maximum Test. Maximal strength (1-RM) was determined according to the guidelines and recommendations described by (Brzycki, 1993) for untrained subjects on analysis of strength using 1-RM. It was determined indirectly by applying the equation for predicting 1-RM based on reps-to-fatigue [24] for the following tests: bench press, leg extension, leg curl, lat pulldowns, overhead press, and shoulder press. Two preliminary series were carried out, an initial one to control the technical execution of the movement and a warm-up series with an approximate load of from 30 to 50% of the perceived 1-RM, the resistance being low enough to allow subjects to complete reps easily, separated by a one-minute rest interval. Two to three attempts were then made with 10–20% increments of the load, separated by rest intervals of 3–5 min. The 1-RM test ended when the players achieved a value less than or equal to five repetitions in each exercise. The same researcher evaluated 1-RM attempts in all exercises, ensuring that each athlete performed the full range of motion.

Countermovement Jump (CMJ). To provide information about the reactive strength of the lower limbs, a double leg vertical CMJ without arm swing (i.e., with the hands on the hips) was performed on a contact time platform (Newtest Powertimer 300-series, Newtest Oy, Tyrnävä, Finland) according to the established protocol [25]. During the test, an investigator confirmed the correct knee angle (90°). Each player performed two maximal attempts, interspersed with 45 s of passive recovery, and the maximum height (cm) determined by flight time was recorded.

Squat Jump (SJ). To provide information about leg power performance, an SJ from a stationary semi-squatting position (90°) was performed on a contact time platform (Newtest Powertimer 300-series, Newtest Oy, Tyrnävä, Finland) according to the established protocol [26]. Each player performed two maximal attempts interspersed with 45 s of passive recovery. The maximum height (cm) determined by flight time was recorded.

Abalakov test (ABK). In the countermovement jump with arm swing (Newtest Powertimer 300-series, Newtest Oy, Tyrnävä, Finland), each player performed two maximal attempts interspersed with 45 s of passive recovery, and the maximum height (cm) determined by flight time was recorded.

Isometric handgrip strength (HD). Handgrip strength was measured in the dominant and non-dominant hand. Players performed the test with the test arm fully extended in the vertical axis and not touching the body. A portable hand dynamometer Smedley III T-18A (Takei, Tokyo, Japan) was used for handgrip strength measurement. The hand dynamometer has a range between 0 and 100 kg, with 0.5 kg increments and an accuracy of ±2 kg. Each subject made two attempts of maximal isometric contractions for 5 s with each hand. Rest time between each attempt was 2 min. The dynamometer was adjusted to the participant’ s hand and the best of two maximal trials from each hand was used in data analysis.

Sit and Reach (SaR). The classic sit and reach test was performed to measure the flexibility of the lower back and hamstrings. The test involved sitting on the floor with legs stretched out straight ahead. The soles of the feet were placed flat against the box. Both knees were locked and pressed flat to the floor—the tester assisted by holding them down. With the palms facing downwards, and the hands-on top of each other or side by side, the subject reached forward along the measuring line as far as possible, holding that position for one to two seconds while the distance was recorded in cm.

10 × 5 m Shuttle Test. This test was performed to measure agility (accelerations and decelerations) with change in direction. Time was recorded using a photocell gate (Newtest Powertimer 300-series, Newtest Oy, Tyrnävä, Finland) and software (Newtest PowertimerPC software 2.0 for windows), with an accuracy of ±0.001 s. Timing was activated and stopped when the player passed through the gate.

### 2.4. Statistical Analysis

Exploratory data analysis included mean and standard deviation descriptive statistics, searching for outliers and assessing the normality of distribution by means of the Shapiro–Wilk test. Levene’s test was used to confirm the equality of variances. Student’s *t*-test was used to determine the possible differences in each variable according to players’ gender. Effect sizes (d) were estimated by calculating the 95% confidence intervals for Cohen’s d and interpreted as follows [26]: Trivial (<0.21), Small (0.2 to <0.60), Moderate (0.60 to <1.2), Large (1.2 to <2.0), Very Large (2.0 to <4.0), and Extremely Large (≥4.0). The significance level was set at *p* < 0.05. Data analyses were performed using the SPSS statistical software package (version 25.0; SPSS, Inc., Chicago, IL, USA).

## 3. Results

**Figure 1:**
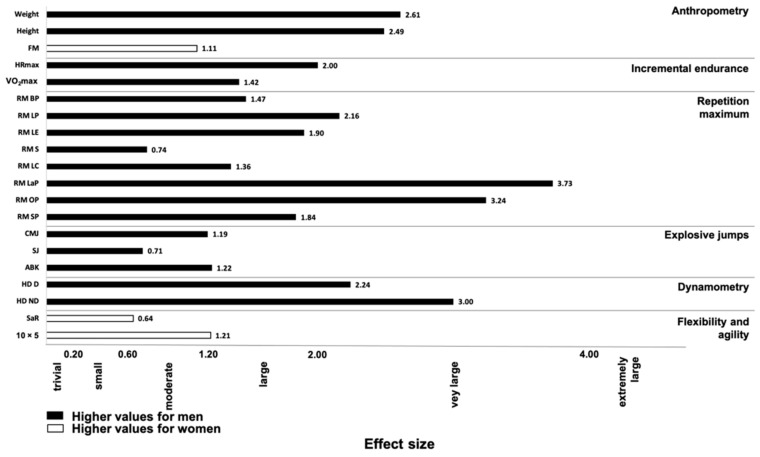
Effect sizes for the gender differences in physical parameters of professional padel players. FM: fat mass; RM: one repetition maximum; BP: bench press; LP: leg press; LE: leg extension; LC: leg curl; LaP: lat pulldowns; OP: overhead press; SP: shoulder press; CMJ: countermovement jump; ABK: Abalakov; HD: hand dynamometer; D: dominant; ND: non-dominant; SaR: sit and reach.

**Figure 2:**
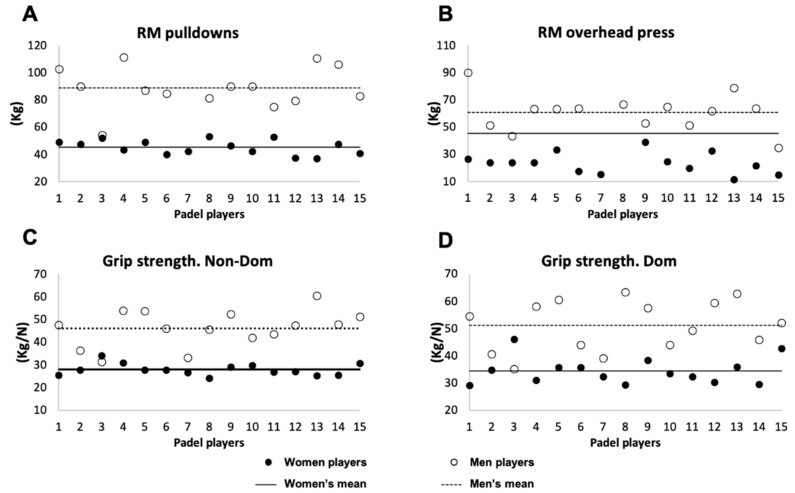
Mean and individual values in the four largest measurements concerning gender differences. RM: one repetition maximum. Non-Dom: non-dominant; Dom: dominant. Kg: kilogram. (**A**) one repetition maximum pulldowns; (**B**) one repetition maximum overhead press; (**C**) grip strength Non-dominant arm; (**D**) grip strength dominant arm.

The results of the test performance are presented in Table 2. The men players recorded higher values in VO_2_ max than the women (*p* < 0.001). In all the one maximum repetition tests, the men players showed significantly higher values than the women (*p* < 0.001; ES: from 1.36 to 3.73), except in the 1-RM S test (*p* = 0.205; ES: 0.74) (Figure 1). Moreover, men players jumped higher in all tests (CMJ, SJ, and ABK), although just CMJ and ABK showed significant differences (*p* = 0.002; ES: 1.19 and *p* = 0.01; ES: 1.22, respectively) (Figure 1). No significant differences were found in the SaR test (*p* > 0.05), although men players obtained significantly lower scores in the agility test than women players (*p* = 0.002; ES: 1.21). Finally, Mean and individual values in the four largest measurements concerning gender differences are shown in Figure 2

## 4. Discussion

The main objective of this study was to examine the fitness characteristics of professional padel players and to determine differences in physical performance regarding players’ gender. This is the first study to evaluate physical fitness (e.g., VO_2_ max, maximal dynamic strength, hand grip or explosive jumps) in professional men and women padel players. Such knowledge is essential to identify determinant factors in game performance, competitive success and injury prevention in padel.

A few studies have examined physical characteristics related to padel players [11,12,27]. Our results showed that men players had higher values in terms of weight, height and VO_2_ max than women players, and lower values of fat mass percentage. Fat mass results are similar to those obtained in other racket sport studies [12,27,28,29], where the range obtained was between 15–19% in women players and 12–15% in men. It seems that the energy requirements of padel are closer to those reported previously for tennis [30]. Currently, Campa et al. [31] developed a cross-sectional observational study on 1556 athletes, sorted into three groups: endurance, velocity-power, and team sports, obtaining similar results to ours for the velocity-power group, which included tennis.

The VO_2_ max results are similar to those of other racket sports, such as tennis [29,30,32], indicating that padel provokes very similar physiological demands and adaptions. The same results were obtained by Carrasco et al. (2011) when evaluating twelve top-level padel players [33]. However, several studies observed higher values of VO_2_ max in squash players, where game intensity is higher than in padel [34,35].

The modern game of padel has become a sport where strength and explosive actions in upper and lower limbs have gained great relevance for success, for example, in short-distance sprints and changes in direction [13,14,36]. In this regard, the smash stroke is the game action producing the highest percentage of winners in padel [7,33], and greatly influences the match outcome. A recent study reported that men players can finish the point with a smash from 7 m to the net [37], while women perform it closer to the net. These authors attribute these results to anthropometric and strength differences, which reflects the importance of strength in this sport. Due to the lower strength and power levels of women players compared with men, we can suggest that performance in women players could be more related to aerobic fitness. In addition, a longer playing time and total time has been observed in women over men players, as well as in the number and type of stroke [6], using more trays and less flat and topspin smashes (powerful strokes) than men [37].

In addition, power, is probably the most important factor in determining success in many sports [18]. In men’s padel players, the obtained results are lower than those found in other racket sports [38]. It is likely that, in tennis, strength may be more determinant, and used in actions more frequently than in padel. Therefore, the power developed in serve and baseline strokes in a tennis match is greater than in padel, where the smash is the most explosive action related to success.

Significant differences have been observed between genders in almost all fitness parameters, especially in the upper limbs, in both maximal dynamic and explosive strength. Previous research showed that men perform more explosive shots and jumps and over-head strokes, such as powerful smashes, while women’s game pace (shots/sec ratio) is slower [37,39]. In light of these findings, coaches and players should consider incorporating upper limb strengthening exercises in their training routine. Regarding explosive jumps, men and women padel players showed a similar CMJ performance to tennis players [18,20], but lower than those of badminton players [40], with higher values in the men in CMJ (32.67 vs. 24.33) and ABK (39.70 vs. 29.29). Unlike tennis and badminton, the padel groundstroke appears to be a low-power demanding action, in which accuracy and anticipation skills prevail [40].

On the other hand, hand-grip dynamometry showed higher values in padel players than in under-18 tennis players [18], probably due to the predominance of short, fast strokes in padel. Grip strength has been demonstrated to be a good indicator of service speed in junior tennis players [41]. This parameter could be very interesting to evaluate in padel players, where a lot of actions require high values of grip strength, such as smashes or volleys. Finally, padel is an intermittent sport, including continuous changes in direction, where agility could be an important factor related to performance. The game demands fast reactions, leg and whole-body movements and an ability to change direction quickly. The results obtained in the 10 × 5-metre shuttle run are similar to those of other racket sports, such as squash [42], indicating than padel could have a high demand for these movements, directly related to performance.

This study was strengthened by the novelty and the relevance of the sample (world-class padel players) and the multifaceted performance evaluation. Furthermore, the use of field tests has been shown to enhance replicability and applicability to the conditioning training sessions. Nonetheless, there are several limitations that must be acknowledged. It is worth noting that we used traditional methods to evaluate 1RM of the different exercises. Alternatively, the 1RM could be accurately estimated by measuring the barbell/machine velocity (i.e., velocity-based approach), thus avoiding the risk of injury and fatigue status related to 1RM tests [43,44]. Additionally, shoulder internal/external rotation strength tests or shoulder ROM were not used. There are different studies in racket sports that show a relationship with hitting speed as well as risk of injury due to imbalances [45,46]. Moreover, field tests such as medicine ball throws or smash velocity were not included, and players’ hand-dominance was not considered.

## 5. Conclusions

Padel is a high-intensity intermittent sport, where players perform short, high-intensity sprints, with changes in direction, hitting high balls and jumping to make different strokes throughout the game. This is the first study to analyse the physical fitness characteristics in professional padel players. The results of this study show that padel players presented anthropometric and physical differences according to gender. They show higher explosive strength values in men players than in women players, in line with the demands of the competition, with shorter point lengths and more explosive strokes (smashes) in the men than the women. As one of the most important principles in training is individualisation, physical trainers now have cardiorespiratory, strength and agility values that can be considered for optimising training and testing procedures for professional padel players.

## Figures and Tables

**Table 1:** Characteristics of the professional women and men padel players.

	Women M (SD)	SE	Men M (SD)	SE	df	*p*
Training per week (hours)	24.1 (3.3)	0.86	23.5 (3.7)	1.02	0.452	0.655
Experience	10.8 (3.5)	0.88	8.1 (3.1)	0.79	2.298	0.029
Age	30.0 (4.2)	1.06	27.4 (6.8)	1.76	1.316	0.198
Weight (kg)	59.9 (4.7)	1.18	78.2 (8.5)	2.21	−7.451	<0.001
Height (cm)	166.6 (4.8)	1.2	177.9 (4.0)	1.02	−7.108	<0.001
FM (%)	19.48 (5.6)	1.5	13.53 (4.83)	1.25	3.07	0.005

FM: fat mass; M: mean; SD: standard deviation; SE: standard error of mean; *p*: significant difference.

**Table 2:** Differences in physical test by gender.

Variables/Test	Women M (SD)	SE	Men M (SD)	SE	df	*p*
**Physical**						
HRmax (lat·min^−1^)	187.53 (9.83)	2.53	185.35 (10.47)	2.80	0.577	0.569
VO_2_ max (mL/kg/min)	46.77 (4.57)	1.22	55.43 (7.04)	1.82	−3.895	<0.001
**Repetition maximum (1-RM)**						
1-RM BP	38.89 (9.87)	2.54	66.40 (24.92)	6.66	−4.075	<0.001
1-RM LP	88.78 (16.3)	4.52	143.22 (31.94)	10.1	−5.333	<0.001
1-RM LE	84.41 (20.37)	5.26	142.35 (36.62)	9.46	−5.354	<0.001
1-RM S	135.37 (31.1)	8.31	179.23 (73.87)	19.07	−2.056	0.205
1-RM LC	72.82 (20.65)	5.33	131.78 (56.11)	14.49	−3.819	<0.001
1-RM LaP	45.45 (5.43)	1.4	89.09 (15.4)	4.12	−10.323	<0.001
1-RM OP	23.57 (7.7)	2.06	60.82 (13.78)	3.68	−8.828	<0.001
1-RM SP	36.94 (9.19)	2.37	105.21 (52.12)	14.46	−4.999	<0.001
**Explosive jumps**						
CMJ (cm)	24.33 (5.4)	1.39	32.67 (7.98)	2.06	−3.349	0.002
SJ (cm)	21.55 (4.65)	1.2	26.67 (8.77)	2.26	−1.998	0.135
ABK (cm)	29.29 (5.35)	1.38	39.07 (9.68)	2.5	−3.425	0.010
**Dynamometry**						
HD D (kg)	33.72 (5.64)	1.41	51.14 (9.19)	2.37	−6.41	<0.001
HD ND (kg)	27.41 (3.33)	0.83	46.19 (8.06)	2.08	−8.581	<0.001
**Flexibility and agility**						
SaR (cm)	34.32 (7.59)	2.03	28.27 (10.39)	2.68	1.779	0.087
10 × 5 (s)	17.62 (1.15)	0.31	16.16 (1.2)	0.31	3.345	0.002

M: mean; SD: standard deviation; SE: standard error of mean; *p*: significant difference; 1-RM: one repetition maximum; BP: bench press; LP: leg press; LE: leg extension; LC: leg curl; LaP: lat pulldowns; OP: overhead press; SP: shoulder press; CMJ: countermovement jump; ABK: Abalakov; HD: hand dynamometer; D: dominant; ND: non-dominant; SaR: sit and reach.

## Data Availability

The data presented in this study are available on request from the corresponding author.
